# High Throughput Fabrication of Flexible Top-Driven Sensing Probe

**DOI:** 10.3390/polym14235124

**Published:** 2022-11-24

**Authors:** Fei Li, Xi Liu, Wensheng Wang, Haoyan Xu, Wenlong Song, Zhuangzhi Sun

**Affiliations:** 1 Province Key Laboratory of Forestry Intelligent Equipment Engineering, College of Mechanical and Electrical Engineering, Northeast Forestry University, Harbin 150040, China; 2 Key Laboratory of Bio-Based Material Science & Technology, Ministry of Education, Northeast Forestry University, Harbin 150040, China

**Keywords:** smart materials, flexible probe, top-driven sensing, thrombus localization

## Abstract

In this work, considering the current status of conservative and complicated traditional thrombosis treatment methods, a kind of flexible intelligent probe (FIP) with a top-driven sensing strategy is proposed to realize the expected function of thrombosis accurate localization in a liquid flow environment. After throughput fabrication, we find that the FIP has excellent electrical conductivity and mechanical properties. Notable, our FIP with the principle of piezo-resistive sensing has a quasi-linear sensitivity (approx. 0.325 L per minute) to flow sensing in the low flow velocity range (0–1 L per minute). Via the well-designed magnetically driven method, our FIP has a maximum deflection output force of 443.264 mN, a maximum deflection angle of 43°, and a maximum axial force of 54.176 mN. We demonstrate that the FIP is capable of completing the specified command actions relatively accurately and has a good response to real-time sensing feedback performance, which has broad application prospects in thrombus localization detection.

## 1. Introduction

Smart materials, including electroactive polymers, shape memory, electrostrictive polymers, etc., are regarded as well-organized functional materials that exhibit sensing changes and moderate response when exposed to environmental stimuli (e.g., light, heat, electricity, magnetism, stress, or chemicals) [1,2,3,4,5]. Smart materials have been widely studied in flexible electronics, including sensors [6,7,8], actuators [9,10,11], and generators [12,13,14] due to their excellent flexibility, wettability, responsiveness, biocompatibility, bioactivity [15,16,17], and their potential in the biomedical field [18,19,20]. However, although the study on smart materials is still in the laboratory stage, the successful application practice of some extreme environments (underwater, vacuum, etc.) and special fields (medical treatment, health monitoring, etc.) has become a new spring for smart materials [21,22,23,24,25,26,27,28,29].

Cardiovascular disease is a common disease that seriously threatens the health of human beings [30,31,32]. Interventional therapy is an effective method to treat cardiovascular disease, which puts higher requirements on the precision of equipment and instruments [33,34,35]. At present, relevant reports on smart materials serving the treatment of cardiovascular disease mainly focus on nanomaterial-based immunosensors for accurate diagnostics [36,37], cardiovascular implants [38], antidiastole for cardiovascular disease [39], as well as stent designs and coronary applications [40], which present great potential in the future medical market. However, in the thrombus treatment operation, a rapid thrombus location when intervened in a fluid blood environment is required, and then the flexible top-driven strategy is used to realize the targeted thrombus removal. Therefore, there is an urgent need for an intelligent thrombus localization strategy to assist the surgeon in diagnostic procedures and to reduce the risk of harm to the surgeon. In addition to the requirement that the probe can work in a liquid flow environment, it is more important that the smart probe can accurately locate the thrombus.

Herein, focusing on the current status of the thrombotic disease treatment, this paper proposes a flexible intelligent probe (FIP) with the function of top-driven sensing in a simulated blood flow environment. After throughput fabrication, the deflection output force of the 3 mm and 8 mm samples with a magnetic particle concentration of 40 wt% and molded in a 90° magnetic field reached a maximum of 443.264 mN and 43°, respectively, while the 8 mm sample with a magnetic particle concentration of 40 wt%, formed in a 0° magnetic field reached a maximum performance axial output force of 54.176 mN. This work is of great significance for the application of multi-functional flexible smart devices and provides inspiration for the development of thrombus removal devices.

## 2. Results and Discussion

### 2.1. Model and Characterization Analysis of the Flexible Intelligent Probe

The design of the FIP is oriented towards the working environment within the blood vessels. The FIP should first come with the health and safety of the human body while taking into account being non-toxic and harmless. Simultaneously, due to the narrow working space causing the destruction to the blood vessels, the working characteristics of high sensitivity, fast response speed, stable working conditions, etc. are possessed to guarantee the accuracy of the work. Additionally, a long working life of the FIP is also ensured. 

**Figure 1:**
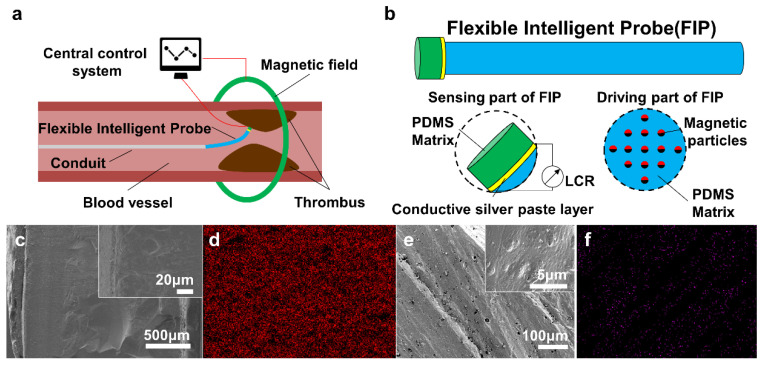
Model and characterization of the flexible intelligent probe (FIP). (**a**) Working model diagram of FIP, (**b**) Composition model diagram of FIP, (**c**) SEM images of cross-section of the piezo-resistive sensing part, (**d**) Element distribution of the piezo-resistive sensing part, (**e**) SEM images of cross-section of the magnetic driving part; (**f**) Element distribution of the magnetic driving part.

Based on the above considerations, our FIP adopts the principle of piezo-resistive sensing (PRS) and magnetic driving (MD), and the schematic diagram of the design model is shown in Figure 1a. The FIP is mainly composed of a PRS part and an MD part, which are connected through a conductive silver paste layer. The PRS part of the FIP is located at the front end, whose deflection is driven by the MD part. The former is to detect the blood flow rate and the position of the thrombus, and transmit the electrical signal in real time. The latter is to drive the probe and the catheter at the rear end in an in-and-out and deflecting motion, and approach the thrombus position to achieve the relevant operation. As shown in Figure 1b, the PRS part is fabricated from a poly dimethyl siloxane (PDMS) film spin-coated with conductive silver paste, while the MD part is composed of PDMS solution incorporating rubidium iron boron magnetic particles, which is formed at high temperature under a specific directional magnetic field.

To deform elastically in response to external environmental stimuli, it showed that the conductive silver paste layer is closely bonded to the PDMS substrate, and the cross-section of the PRS part is homogeneous in texture (Figure 1c). Furthermore, the composition analysis of the X-ray presented that the distribution of silver elements is very uniform, indicating the presence of a conductive silver paste layer on the PRS part (Figure 1d). This enables the normal transmission of electrical signals for piezo-resistive sensing. In the MD part, the dense PDMS matrix has a fine pore structure (Figure 1e), which provides good storage for the encapsulated and fixed neodymium ferrum boron magnet (NdFeB) magnetic particles, avoiding interference with the magnetic response of driving motion. Here, the neodymium element in NdFeB was selected to characterize the internal magnetic particle distribution by localization. The particles showed a chain structure parallel to the molded magnetic field of 45° (Figure 1f). At low content of NdFeB, the chain structure is short and thin, and the number of the chain structures is small, but the spacing between adjacent chains is large. On the contrary, the law is the opposite. The spacing between adjacent chains decreases, and sometimes, the chain structures are interlaced.

### 2.2. Sensing Mechanisms and Working Performance of the FIP

**Figure 2:**
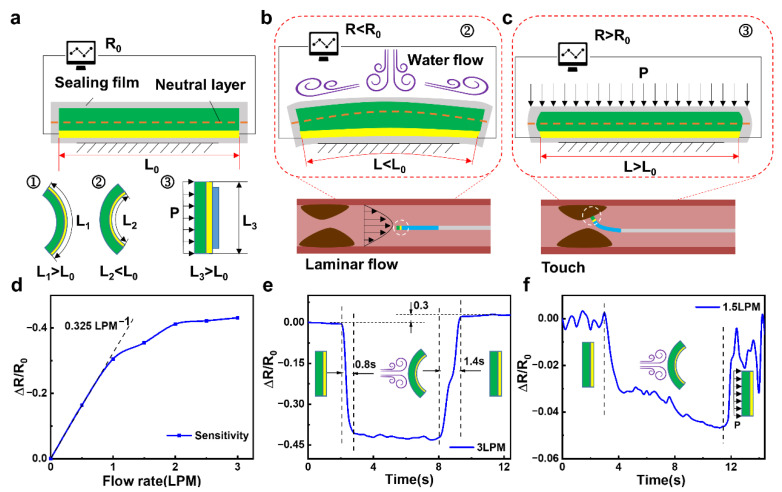
Sensing mechanism and performance of the FIP: (**a**) Deformation sensing mechanism of the FIP, (**b**) Velocity sensing mechanism of the FIP, (**c**) Positioning sensing mechanism of the FIP, (**d**) Sensitivity curve of the FIP, (**e**) Response time curve the FIP, (**f**) Distance sensing curve of the FIP.

As shown in Figure 2a, the conductive silver paste connected with wires and PDMS substrate are encapsulated in a waterproof layer. The entire area of the PRS part is slightly larger than the area of the MD part, in order to better sense the changes in low-velocity water flow. When the PRS part is deformed, the length of the neutral layer does not change. However, the conductive silver paste is located on one side of the neutral layer, so it is extremely simple for deformation to occur. According to the resistance formula of *R = ρL/S*, where *ρ* is the resistivity, *L* is the length along the circuit conduction direction, *S* is the cross-sectional area perpendicular to the circuit conduction direction. When the conductive silver paste is deformed, it will cause its own resistance length *L* to change, thus causing a change in resistance value (Figure S1).

The sensing detection mechanism of the flow velocity is shown in Figure 2b. The impact of the blood flow will produce a compressive deformation when the FIP enters. Since the area of the PRS part is larger than the area of the MD part, the two ends of the PRS part are in a free state and will compress backward upon impact, causing the entire surface of the PRS part to form a shape with the least possible resistance. This shape, in turn, causes the conductive silver paste inside it to be compressed so that its length *L* is less than the initial length *L*_0_, resulting in a lower resistance. The faster the flow rate in the blood vessel, the stronger the impact effect on the PRS part. Also, the more pronounced the deformation of the PRS part, the more pronounced the change in resistance. Indirect sensing of blood flow velocity is achieved through this phenomenon. Simultaneously, the sensing detection mechanism of the thrombus position is shown in Figure 2c. When the FIP touches the thrombus, which forms a kind of splint with the fixed surface of the PRS part, and it will squeeze the PRS part from both sides. This results in a compressive elongation of the PRS part, where the length *L* of the conductive silver paste layer is greater than the initial length of *L*_0_, resulting in an increase in resistance.

In conjunction with the previous mechanics of sensing blood flow velocity, the presence of a thrombus causes a narrowing of the blood vessel, which leads to an increase in flow velocity in the vicinity of the thrombus, assuming that the human blood flow is constant. As the FIP slowly approaches the thrombus from the distal end, the flow velocity tends to increase within a certain range, at which point the resistance of the piezo-resistive sensing component gradually decreases, reaching a minimum value when the FIP is close to the thrombus. When the FIP touches the thrombus, the resistance of the PRS part increases rapidly. The dynamic process of this feature is monitored to determine if a thrombus is present, and the related laboratory bench is shown in Figure S2.

The flow sensing performance mechanism can be valued by the sensitivity equation of *S = d(*Δ*R/R*_0_*)/dv*, where Δ*R* is the changing resistance value, *R*_0_ is the initial resistance value and *v* is the flow velocity. A curve of the relative resistance change of the PRS part with the flow velocity is measured and is shown in Figure 2d, and the sensing performance curves for different flow rates are shown in Figure S3. In the low flow rate range (0–1 L per minute (LPM)), the PRS part shows a quasi-linear sensitivity to the flow rate of approximately 0.325 LPM. The higher sensitivity in the low flow rate range is mainly due to the good elastic deformation of the PDMS substrate, which deforms well in a linear fashion and causes a change in resistance. As the flow rate continues to increase, the sensitivity of the device gradually is decreased due to the degree of elastic deformation beyond the linear deformation, which is influenced by the elastic resistance of the PDMS material but is still able to differentiate sensing for different flow rates.

Figure 2e shows the response speed and recovery characteristics of this PRS part for flow rate detection at 3 LPM. The response time is measured to be approximately 0.8 s for one cycle of flow rate application and release, demonstrating a relatively fast response rate. In contrast, the response time for detection at flow rate withdrawal is approximately 1.2 s. The sensing response at different flow rates shows a recovery response time that is higher than the detection sensing time due to the fact that at high flow rates, the PDMS substrate is out of its elastic deformation state and returns to its original form more slowly and takes longer. During a cycle of flow application and release, when the flow rate is removed, the resistance does not return to its initial value but becomes slightly higher than the initial resistance *R*_0_, which is approximately 1.3 times the initial resistance value. This is due to a certain inertia in the recovery of its own deformation after the withdrawal of the flow rate, resulting in a recovery of the deformation even after reaching its original shape, making the resistance value larger. 

In order to investigate the distance sensing performance of the thrombus, the FIP was used to slowly approach (2 mm/s) the simulated thrombus at a constant flow rate (1.5 LPM). As shown in Figure 2f, the resistance value increases as the FIP is brought closer and closer due to the presence of the simulated thrombus, which causes a change in the actual internal tube diameter, resulting in a significant increase in the flow velocity around the thrombus. This increase in flow velocity causes the PRS part to deform itself, which in turn causes a change in resistance value. The faster the flow rate, the more the PRS part is bent backward, and the more the conductive silver paste layer is squeezed, resulting in a gradual reduction in resistance by approximately 0.04 of its own resistance value. This change in resistance allows for a good approximation of the thrombus location. 

### 2.3. Driving Mechanisms and Performance Testing of the FIP

**Figure 3:**
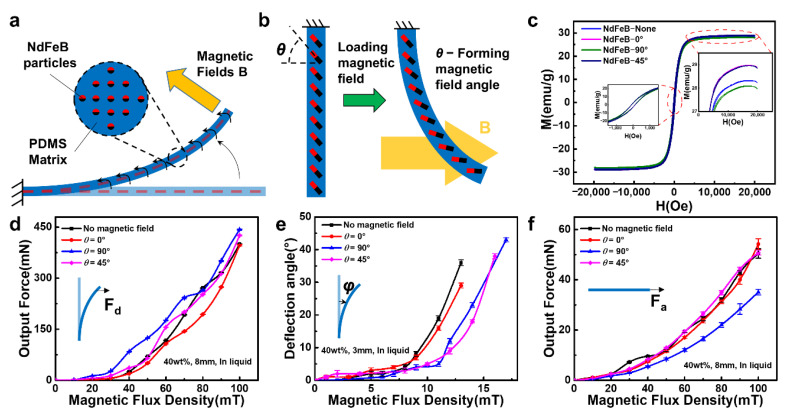
Driving mechanism and performance of the FIP: (**a**,**b**) Driving mechanism of the FIP, (**c**) Hysteresis line of the MD part, (**d**) Deflection output force curve of the FIP, (**e**) Deflection angle curve of the FIP, (**f**) Axial output force curve of the FIP.

The motion mechanism of the MD part of the FIP is relatively straightforward and is driven by the interaction between the driving magnetic field and the hard magnetic particles (NdFeB particles) dispersed in the polymer matrix (PDMS matrix) (Figure 3a). The PDMS matrix containing the NdFeB particles can be divided into multiple magnetic polymer units. In a magnetic field, the driving force can be thought of as a magnetic torque, and the magnetic polymer unit is subjected to the magnetic torque to generate movement and deformation, which ultimately results in the movement of the MD part of the FIP. As the actuating magnetic field is much smaller than the saturation field, the magnetization of the magnetic polymer units (voxels) is independent of the actuating magnetic field. As shown in Figure 3b, as the FIPs are molded under different magnetic fields, the internal magnetic particles are arranged in different directions, which results in different output forces and deflection angles when a directional magnetic field is applied. 

The hysteresis lines of the MD parts molded under different magnetic field orientations are shown in Figure 3c. The differences in values also indicate that the direction of the magnetic field molding has an effect on the magnetic response, which corresponds to the analysis of the mechanical motion properties. The very small coercivity (approx. 105 emu/g) and remanence (approx. 2.51 Oe) indicate that the magnetically responsive flexible actuator is softly ferromagnetic. The area enclosed by the hysteresis lines indicates the energy consumed to magnetize a ferromagnetic substance for one week, which is often converted into heat and consumed. The smaller area of the hysteresis lines of different magnetic field orientations of the flexible actuators above indicates that less energy is required to drive them and that they can be driven at low power, which is beneficial to the design of the equipment. 

The motion performance of the FIP was investigated using an aqueous solution of propylene glycol formulated to the same viscosity as blood. Figure 3d–f and Figures S4–S7 showed the deflection output force, deflection angle, and output force of the magnetically driven part of the FIP located in a liquid environment at different magnetic induction strengths (0°, 45°, 90°). Obviously, the sample molded in a 90° magnetic field has the strongest response to the deflecting magnetic field because its magnetic induction direction is the same as the deflecting magnetic field direction, while the sample molded in the 45° and 0° magnetic fields is less responsive because of the angle with the deflecting magnetic field direction, which leads to a decrease in the output force. If the number of internal magnetic particles in line with the direction of the external deflection field is higher, the output force performance is better, and vice versa.

Meanwhile, the sample molded in a 90° magnetic field has the strongest response to the deflection field (Figure 3e and Figure S6), as its magnetic induction direction is the same as the direction of the deflection field, which makes the initial deflection response extremely fast, but when deflection occurs, the magnetic induction direction of the internal particles becomes angled to the deflection field, and the deflection angle performance is slightly reduced later on. The 45° and 0° magnetic field samples are initially less responsive because of the angle with the deflecting magnetic field, but as the deflection process proceeds, the angle with the deflecting magnetic field is smaller, and there is a higher upward trend later on. As for the free-forming sample without a magnetic field, its internal magnetic particle direction is not fixed, so there is uncertainty in the output angle. If the number of internal magnetic particles in line with the direction of the external deflection magnetic field is higher, the better the deflection angle performance, and vice versa, so it presents an uncertain situation.

In addition, the response to the axial magnetic field is the strongest for the 0° sample, because the magnetic induction direction is the same as the axial magnetic field direction, while the response of the 45° and 90° samples is reduced due to the angle with the axial magnetic field direction, which leads to a decrease in the output force (Figure 3f and Figure S7). If the number of internal magnetic particles in line with the direction of the external axial magnetic field is higher, the output force performance is better and vice versa, so it presents a situation between the sample molded under 0° magnetic field and the sample molded under 90° magnetic field.

### 2.4. Working Process and Simulation Experiments of the FIP

**Figure 4:**
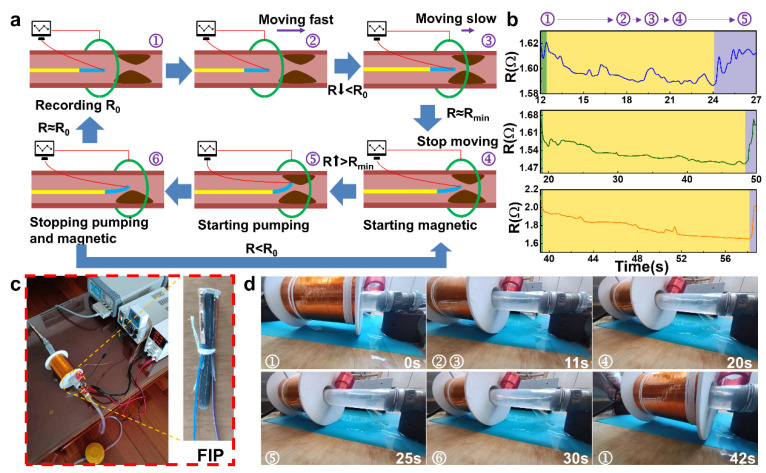
Simulation experiments of the FIP: (**a**) Workflow diagram of the FIP: ① initial state; ②, ③ fast monitoring; ④ intelligent positioning; ⑤ pumping and clearing; ⑥ end and next monitoring, (**b**) Sensing feedback curves in the simulation experiments, (**c**) Experimental testbed for thrombus simulation and real FIP, (**d**) Experimental procedure during simulated thrombus removal.

The overall workflow of the FIP can be described as follows (Figure 4a). Initially, the front piezo-resistive sensing part is operated to set the initial value, and when the sensing signal is stable, the motion coil is energized and activated, sliding forward to drive the FIP forward rapidly. When the monitoring signal changes (the resistance value decreases), the FIP is approaching the thrombus. The control system controls the size of the coil voltage output and the sliding speed by the degree of signal change, with low voltage, low magnetism, and slow deceleration. When the preset minimum resistance value is reached, it is considered to be close to the thrombus, and the motion coil stops supplying power and movement, activating the deflection coil, which deflects the FIP to make close contact with the thrombus. When the sensing signal value changes (rises), the thrombus has been contacted, and the deflection coil maintains the operating voltage at this point, ensuring that the FIP remains in its current form and is able to complete the corresponding operation in a stable manner. At the end of a cycle of corresponding operations, the deflection coil voltage is removed, and the FIP is returned to its natural state, at which point the sensing signal is monitored and compared to the signal in the absence of a thrombus. 

According to the above working mechanism, two working modes are selected in the following cycle. If the signal differs significantly from that in the absence of a thrombus, then the operation is not complete, and the deflection coil is activated to drive the FIP through another operation cycle. However, if the signal is close to the signal in the absence of a thrombus, then the operation is considered complete, and the operation is continued to the next step or the probe is withdrawn to end the operation. 

To demonstrate the working parameters under actual operating conditions, real-time sensing feedback signals of FIP were recorded simultaneously to facilitate a clearer analysis of the sensing signals. Figure 4b showed all three sensing performance curves are consistent with the thrombus characteristic curves of the three working cycles (Figure 4a), indicating that the general location of the thrombus can be detected in all three cycles. This regular change in flow rate, which is the heart pumping blood, was simulated and the sensing signal was also able to respond in the form of resistance fluctuations. Meanwhile, as the distance between the FIP and the simulated thrombus decreases, the flow velocity around the probe increases, and the resistance value decreases. When the simulated thrombus is touched, the PRS part of the probe is elongated by compression and the resistance increases. The real-time sensing feedback performance tests during the actual working conditions provide a good response to the working condition and environment of the FIP.

The construction of the entire vascular simulation environment is shown in Figure 4c. After assembling and commissioning the experimental equipment and devices, the actual working motion state of the FIP was simulated and investigated in Figure 4d. The main focus of this process was to observe and investigate the accuracy of the working attitude of the magnetic driving part of the FIP. The FIP, stimulated by the driving magnetic field, follows the coil in its slow forward motion, reaching the second half of the line at approximately 11 s. At approximately 20 s, the PRS part detects the presence of the thrombus and the drive coil stops moving forward and switches off the drive magnetic field, at which point the FIP is positioned close to the location of the simulated thrombus. The deflection coil is then energized to generate a deflecting magnetic field, which drives the probe to one side and performs a close proximity movement, which is confirmed by the sensing signal. During the entire working process, the FIP is able to complete the specified command action more accurately under the stimulation of the driving and deflecting magnetic fields according to the operation needs. In the future, this kind of throughput smart probe shows great potential market prospects during minimally invasive surgery for positioning and removal of the human thrombus.

## 3. Conclusions

In this work, we propose the design of a flexible intelligent probe (FIP) with a top-driven sensing strategy to realize the function of thrombus location sensing in a liquid flow environment. The sensitivity, response rate, and recovery characteristics of the FIP were analyzed to confirm its sensing performance for both flow velocity and thrombus position. Via the well-designed magnetically driven method, the deflection output force of the 8 mm, 3 mm sample with a magnetic particle concentration of 40 wt% and molded in a 90° magnetic field reached a maximum of 443.264 mN and 43°, while 8 mm, magnetic particle concentration of 40 wt%, formed in a 0° magnetic field with a maximum performance axial output force of 54.176 mN. The workflow of the FIP is described, and the operating position and signal detection performance of the FIP under simulated real-world conditions indicate its promising potential for thrombus location detection. In the future, this cheap and intelligent flexible probe is expected to serve as an important part of the intelligent localization and removal of human thrombus in minimally invasive surgery. In addition, the development of the control algorithm for this intelligent probe is also an effort trend that has to be considered in the application of thrombus removal surgery.

## 4. Experimental Section

The FIP consists of a piezo-resistive sensing (PRS) part and a magnetic driving (MD) part. The process of fabricating the PRS part of the FIP is as follows. Firstly, the PDMS main agent and the curing agent (SYLGARDTM 184 Silicone Elastomer) are weighed in a mass ratio of 10:1, poured into a beaker, mixed for 15 min, then vacuumed to remove air bubbles by spin-coating on a glass plate, and dried in a drying oven at 60 °C to produce the PDMS substrate. The conductive silver paste (made by Luxianzi Co. Ltd in Shenzhen, China) is applied evenly to the surface of the PDMS substrate with a brush, cleaned to remove the excess conductive silver paste and ensure the surface is smooth and flat, then placed in a 60 °C drying oven for 1 h to dry. The dried PDMS substrate conductive silver paste composite layer is cut according to the required size, then the surface ends are fixed with small pieces of copper tape to connect the wires, and then the whole sample is sealed with sealing film to obtain a PRS part of FIP. 

Moreover, the process of fabricating the MD part of the FIP uses the following process, where a 10:1 ratio of PDMS master agent and curing agent (SYLGARDTM 184 Silicone Elastomer) are added to different concentrations of rubidium iron boron magnetic particles, stirred for 15 min until homogeneous, and vacuumed to remove air bubbles. Cylindrical molds of different diameters (3 mm, 5 mm, 8 mm) were made by 3D printing and the PDMS mixture was poured into the molds, which were then dried at 60 °C under different magnetic fields. Here, the angle between the direction of the molding field and the axis of the cylindrical mold is called the angle of the molding field, e.g., the direction of the field is parallel to the axis, i.e., the angle is 0°, which is defined as a 0° molding field; the direction of the field is perpendicular to the axis, i.e., the angle is 90°, which is defined as a 90° molding field; and there is also a 45° molding field with an angle of 45° to the axis. After waiting for the mixed system to dry and form, magnetically responsive MD sections of different concentrations, sizes, and directions of the forming field are prepared. 

The front side of the PRS part of the prepared FIP (the side coated with conductive silver paste) is connected to one end of the MD part. The wire of the PRS part is then fixed to the MD part to prevent the wire from vibrating and causing interference to the PRS part. A detailed fabrication process of the FIP is shown in Figure S8. Here, an efficient process method of high-throughput fabrication [41] was developed that can acquire a large number of different experimental samples in a short period of time. By the above preparation process, a large number of FIPs with different magnetic response properties can be fabricated.

## Data Availability

Not applicable.

## Supplementary Materials

The following supporting information can be downloaded at: www.mdpi.com/article/10.3390/polym14235124/s1. Figure S1: Resistance value of the piezo-resistive sensing part of the FIP in each state, Figure S2: The laboratory bench for the piezo-resistive sensing part of the FIP, Figure S3: Flow rate sensing performance test curves, Figure S4: The laboratory bench for the magnetic driving part of the FIP, Figure S5: The deflection output force of the magnetic driving part of the FIP with different parameters in a liquid environment as a function of magnetic induction strength, Figure S6: The deflection angle of the magnetic driving part of the FIP with different parameters in the liquid environment as a function of magnetic induction, Figure S7: The axial output force of the magnetic driving part of the FIP with different parameters in liquid environment with magnetic induction Figure S8: The detailed production process of the FIP.
