# Supplementary material for: High Throughput Fabrication of Flexible Top-Driven Sensing Probe
Source: Polymers (Basel). 2022 Nov 24;14(23):5124. doi: 10.3390/polym14235124 (PMC9738077; doi:10.3390/polym14235124)
Supplement: Supplementary file 1 [file polymers-14-05124-s001.zip › polymers-1990934-supplementary.pdf]

polymers

Supplementary material

High Throughput Fabrication of Flexible Top-Driven Sensing Probe

Fei Li [1,2,†], Xi Liu [1,2,†], Wensheng Wang [1,2,†], Haoyan Xu [1,2], Wenlong Song [1,2,*] and Zhuangzhi Sun [1,2,*]

[1] Province Key Laboratory of Forestry Intelligent Equipment Engineering, College of Mechanical and Electrical Engineering, Northeast Forestry University, Harbin 150040, China
[2] Key Laboratory of Bio-Based Material Science & Technology, Ministry of Education, Northeast Forestry University, Harbin 150040, China
* Correspondence: wlsong@nefu.edu.cn (W.S.); sunzhuangzhi@nefu.edu.cn (Z.S.)
† These authors contributed equally to this work.

Brief description of what this file includes:

Supplementary Figure S1 Resistance value of the piezo-resistive sensing part of the FIP in each state.

Supplementary Figure S2 The laboratory bench for the piezo-resistive sensing part of the FIP

Supplementary Figure S3 Flow rate sensing performance test curves.

Supplementary Figure S4 The laboratory bench for the magnetic driving part of the FIP.

Supplementary Figure S5. The deflection output force of the magnetic driving part of the FIP with different parameters in a liquid environment as a function of magnetic induction strength.

Supplementary Figure S6 The deflection angle of the magnetic driving part of the FIP with different parameters in the liquid environment as a function of magnetic induction.

Supplementary Figure S7 The axial output force of the magnetic driving part of the FIP with different parameters in liquid environment with magnetic induction.

Supplementary Figure S8 The detailed production process of the FIP.

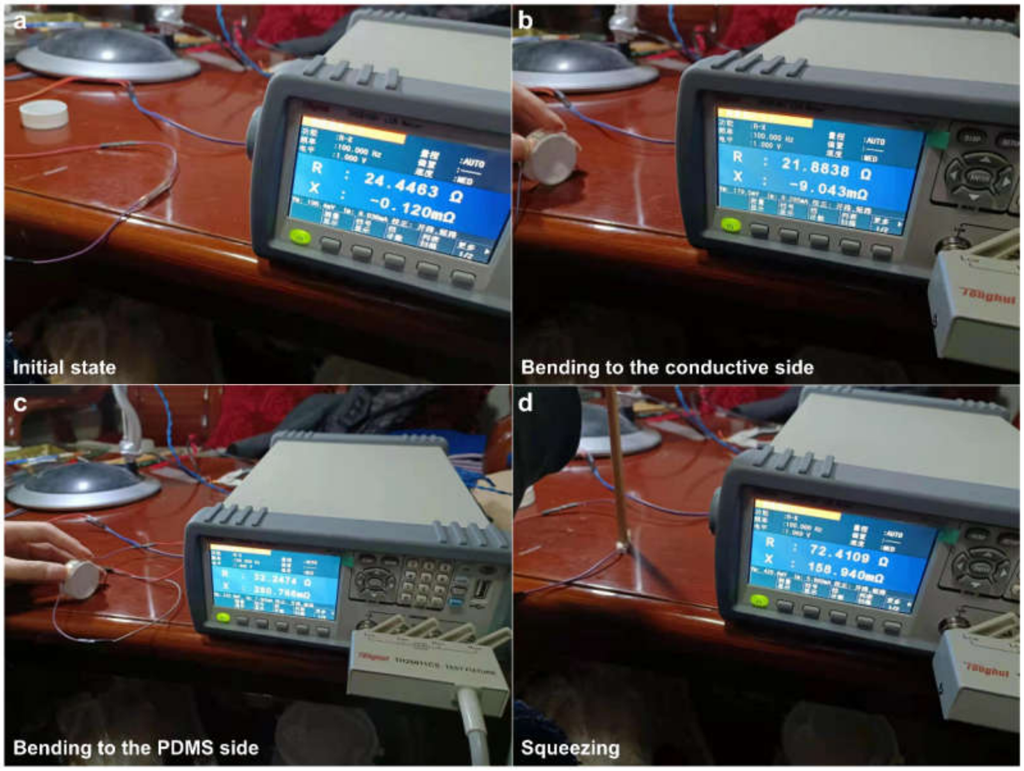

Figure S1. Resistance value of the piezo-resistive sensing part of the FIP in each state: (a) Initial state resistance value of the piezo-resistive sensing part of the FIP, (b) Resistance value of the piezo-resistive sensing part of the FIP when bending towards the conductive side, (c) Resistance value of the piezo-resistive sensing part of the FIP when bending towards the PDMS side, (d) Resistance value of the piezo-resistive sensing part of the FIP when being squeezed.

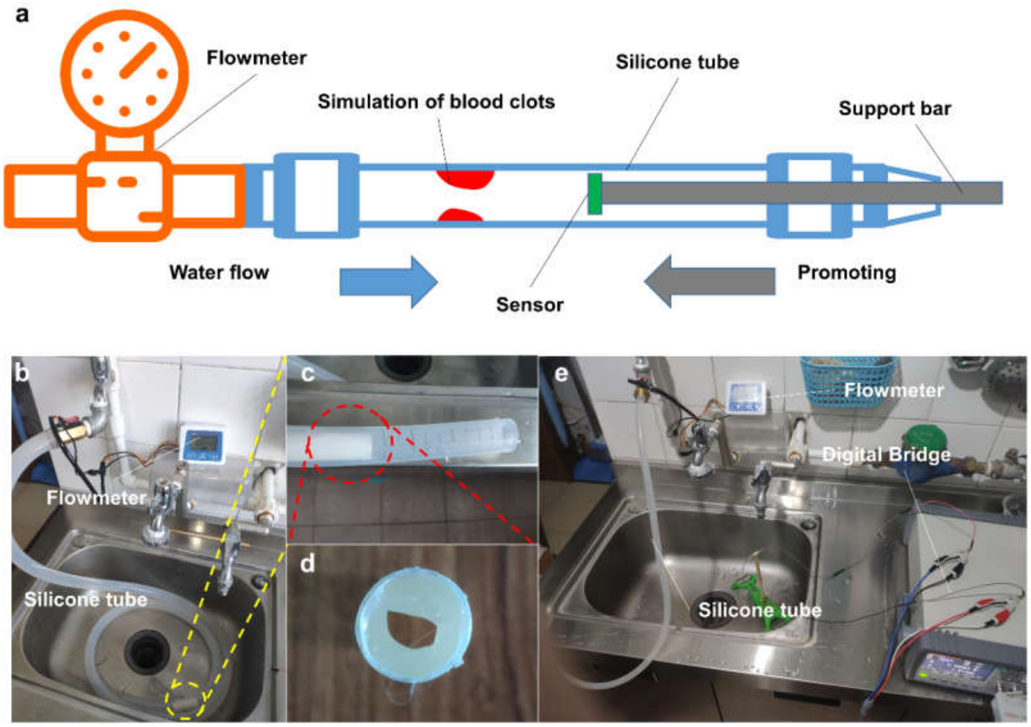

Figure S2. The laboratory bench for the piezo-resistive sensing part of the FIP: (a) Schematic diagram of the laboratory bench of the piezo-resistive sensing part of the FIP, (b) The piping set-up of the laboratory bench, (c) The scale tube for thrombus distance sensing, (d) The thrombus simulation device, (e) The actual measurement of the piezo-resistive sensing part of the FIP.

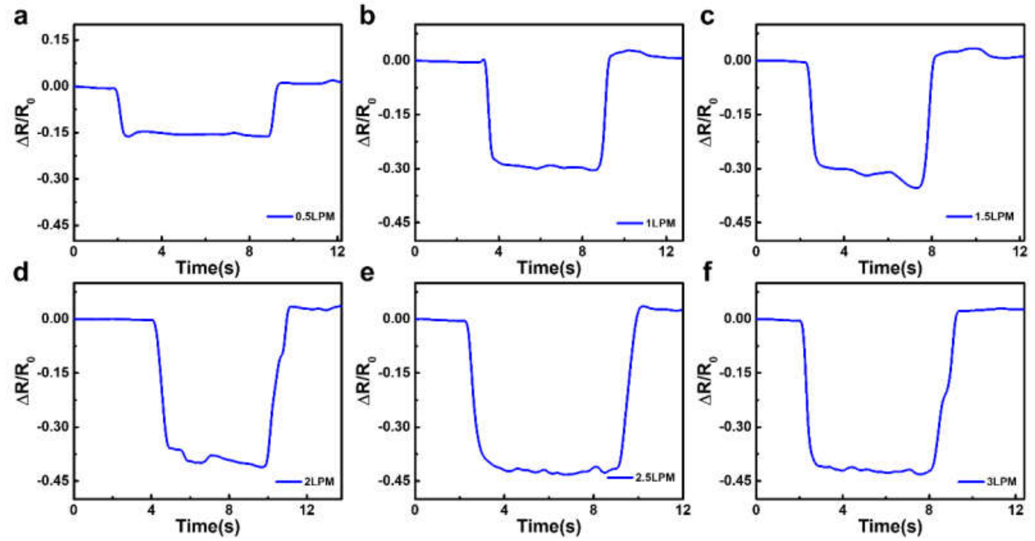

Figure S3. Flow rate sensing performance test curves: (a)-(f) Sensing performance curves for flow rates of 0.5, 1, 1.5, 2, 2.5 and 3 LPM respectively.

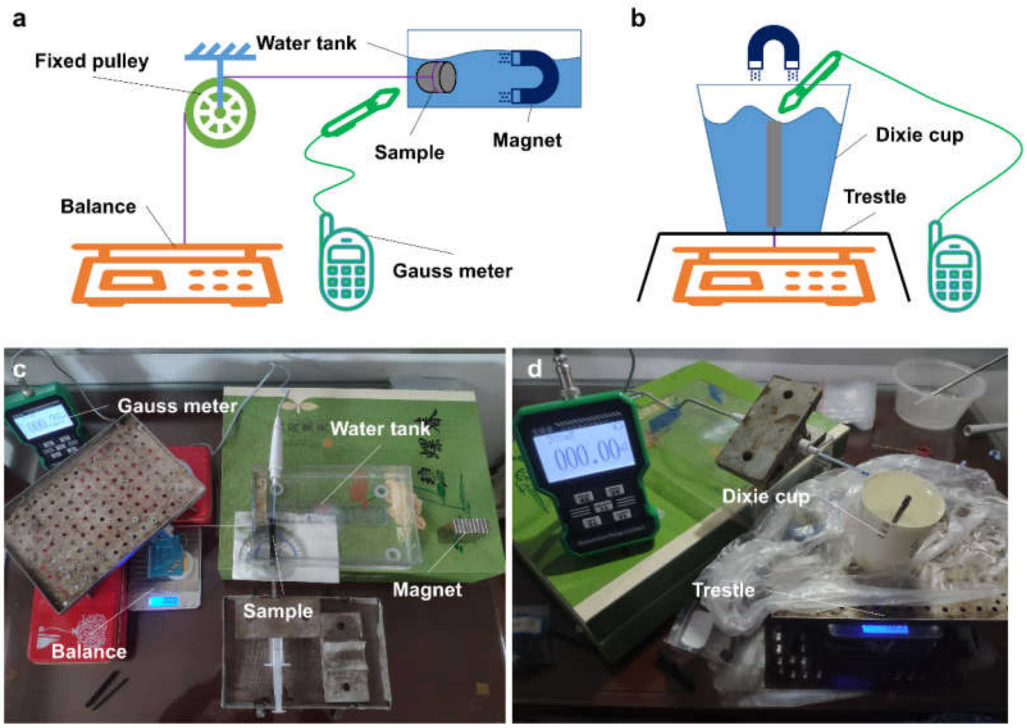

Figure S4. The laboratory bench for the magnetic driving part of the FIP: (a) Schematic diagram of the deflection force and deflection angle laboratory bench, (b) Schematic diagram of the axial force laboratory bench, (c) Physical diagram of the deflection force and deflection angle laboratory bench, (d) Physical diagram of the axial force laboratory bench.

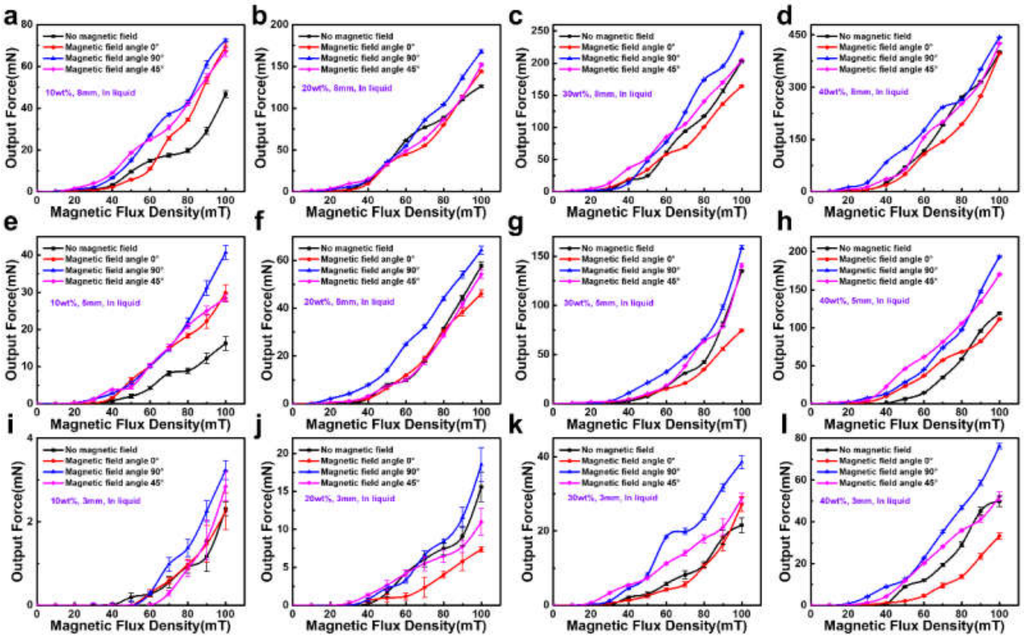

Figure S5. The deflection output force of the magnetic driving part of the FIP with different parameters in a liquid environment as a function of magnetic induction strength: (a)-(d) Curves of the deflection output force of the magnetic driving part of the FIP with 8mm diameter and concentrations of 10 wt%, 20 wt%, 30wt% and 40wt% respectively in a liquid environment as a function of the magnetic induction intensity, (e)-(h) Curves of the magnetic driving part of the FIP with 5mm diameter and concentrations of 10 wt%, 20 wt%, 30 wt% and 40 wt% respectively in a liquid environment. (i)-(l) Curves of the deflection output force of the magnetic driving part of the FIP with a diameter of 3 mm and concentrations of 10 wt%, 20 wt%, 30 wt% and 40 wt%, respectively, in a liquid environment as a function of magnetic induction intensity.

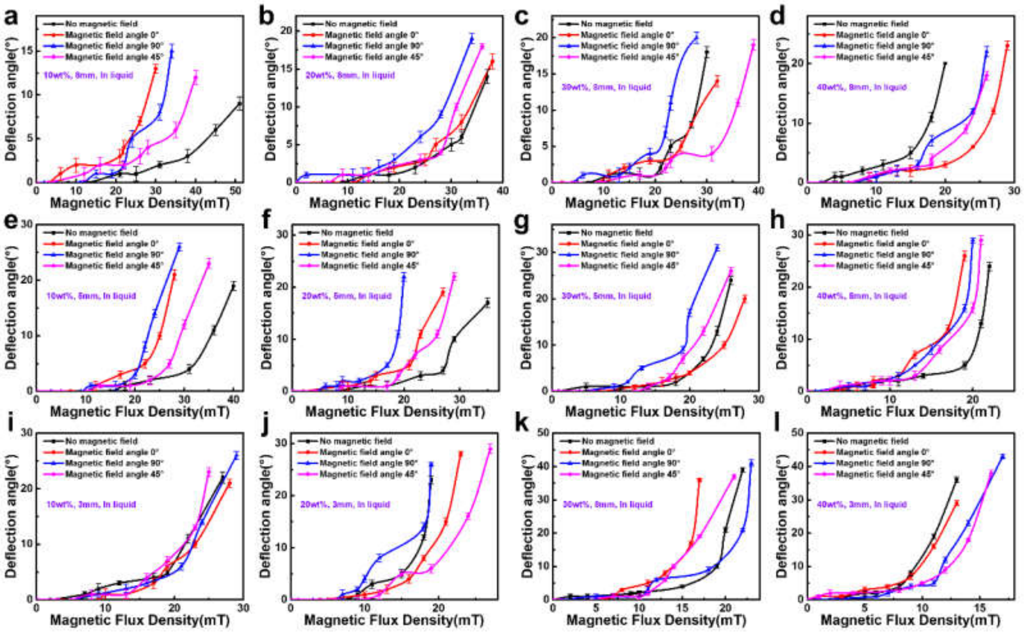

Figure S6. The deflection angle of the magnetic driving part of the FIP with different parameters in the liquid environment as a function of magnetic induction: (a)-(d) Curves of the deflection angle of the magnetic driving part of the FIP with 8mm diameter and concentration of 10 wt%, 20 wt%, 30 wt% and 40 wt% respectively in the liquid environment as a function of magnetic induction, (e)-(h) Curves of the deflection angle of the magnetic driving part of the FIP with 5mm diameter and concentration of 10 wt%, 20 wt%, 30 wt% and 40 wt% respectively in the liquid environment as a function of magnetic induction, (i)-(l) Curves of the deflection angle of the magnetic driving part of the FIP with a diameter of 3mm and a concentration of 10 wt%, 20 wt%, 30 wt% and 40 wt% respectively, in a liquid environment as a function of magnetic induction.

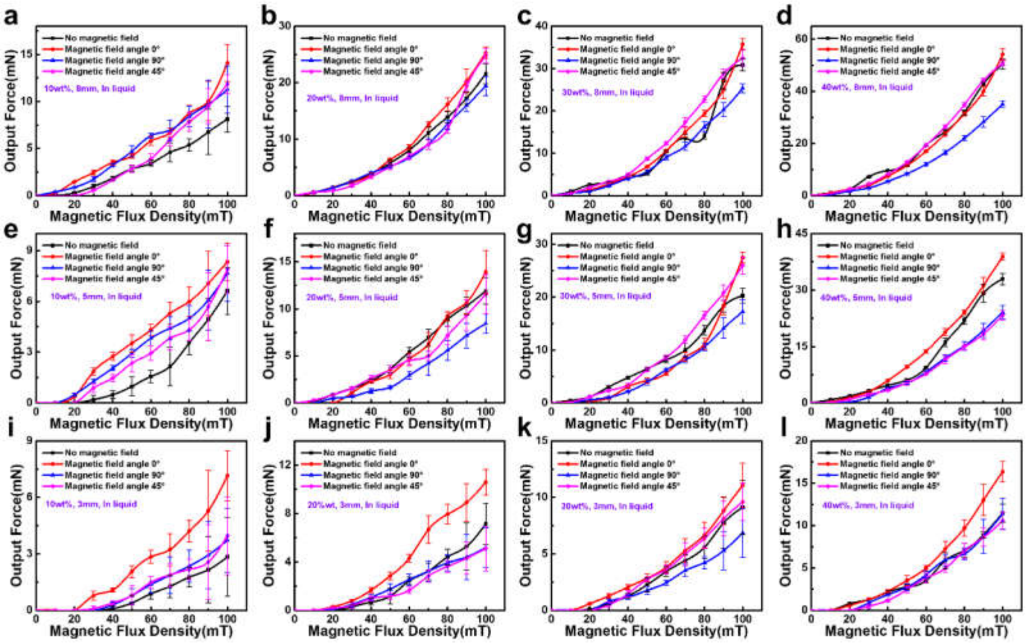

Figure S7. The axial output force of the magnetic driving part of the FIP with different parameters in liquid environment with magnetic induction: (a)-(d) Axial output force of the magnetic driving part of the FIP with 8mm diameter and concentration of 10wt%,

20wt%, 30wt% and 40wt% respectively in liquid environment with magnetic induction, (e)-(h) Curves of the axial output force of the magnetic driving part of the FIP with a diameter of 5 mm and concentrations of 10 wt%, 20 wt%, 30 wt% and 40 wt%, respectively, in a liquid environment as a function of magnetic induction, (i)-(l) Curves of the axial output force of the magnetically driven part of the flexible smart probe with a diameter of 3 mm and concentrations of 10 wt%, 20 wt%, 30 wt% and 40 wt%, respectively, in a liquid environment as a function of magnetic induction liquid environment with magnetic induction intensity.

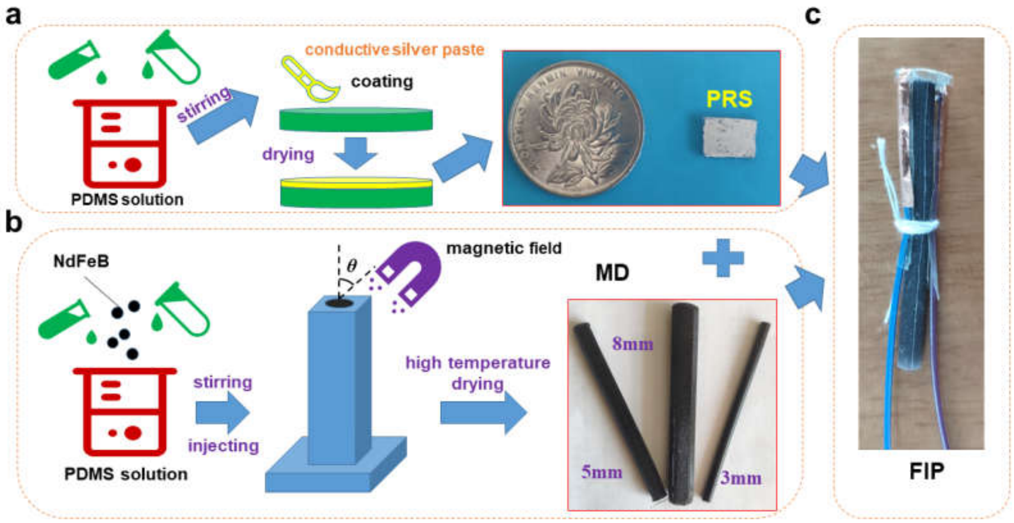

Figure S8. Schematic diagram of the FIP production process: (a) Schematic diagram of the production process of the piezo-resistive sensing part of the FIP, (b) Schematic diagram of the production process of the magnetically driven part of the FIP, (c) Physical view of FIP.
